# Efficacy of Mindfulness-based complementary alternative therapy on physical and mental stress, self-efficacy, and coping styles of patients undergoing breast cancer surgery

**DOI:** 10.12669/pjms.42.1.12519

**Published:** 2026-01

**Authors:** Yemei Wang, Zenghui Ding

**Affiliations:** 1 Yemei Wang Department of Nursing, Hefei Cancer Hospital, Chinese Academy of Sciences, Hefei 230000, Anhui, China; 2 Zenghui Ding Medical Artificial Intelligence, Hefei Institute of Physical Science, Chinese Academy of Sciences, Hefei 230000, Anhui, China

**Keywords:** Breast cancer surgery, Coping styles, Mindfulness-based complementary alternative therapy, Patients’, physical and mental stress, Self-efficacy

## Abstract

**Objective:**

To explore the effect of mindfulness-based complementary alternative therapy on physical and mental stress, self-efficacy, and coping styles of patients undergoing breast cancer surgery.

**Methodology:**

This was a retrospective study. This study included 80 breast cancer patients who underwent surgical treatment in Hefei Cancer Hospital Breast Cancer Center, Chinese Academy of Sciences from May 2023 to December 2024. They were equally allocated to two cohorts (n= 40 per group) using randomization. The control cohort received standard postoperative care, while the experimental cohort was provided with mindfulness-based complementary interventions(MBIs) integrated into conventional nursing protocols. The intervention period was eight weeks. SAS, SDS, VAS, GSES and MCMQ were used to evaluate the effects of the two groups before and after intervention, and the total satisfaction(NSNS) of the two groups was evaluated.

**Results:**

Compared to the control group, the intervention group demonstrated significantly lower levels of anxiety(SAS), depression(SDS), and pain perception(VAS) (all P< 0.05). Post-intervention analyses revealed a notable increase in self-efficacy(GSES) within the intervention cohort relative to controls(P< 0.05). Additionally, the intervention group exhibited enhanced coping strategies, with higher scores in “confrontation,” “avoidance,” and “compromise” domains compared to the control group(P< 0.05). Regarding satisfaction outcomes, the intervention group achieved an overall satisfaction rate of 97.50%, surpassing the control group’s 85.00%(P< 0.05).

**Conclusion:**

Eight weeks of mindfulness-based complementary alternative therapy for patients undergoing breast cancer surgery can effectively relieve postoperative stress responses, enhance their self-efficacy, and optimize coping strategies, which should be promoted in clinical practice.

## INTRODUCTION

Breast cancer, as one of the most common malignant tumors worldwide, is mainly clinically manifested as painless, hard-textured, and irregularly-shaped breast lumps, and some patients may experience all breast pain or discomfort, and, in severe cases, symptoms such as nipple retraction, discharge, or ulceration.^1^,^2^ Since early-stage breast cancer often comes with no obvious symptoms, patients may exhibit abnormalities as the disease progresses, typically presenting at stages-II or III when seeking medical attention.^3^ Currently, the standard treatment for stage-II and III breast cancer in clinical management primarily involves radical surgery and breast-conserving surgery,^4^ which can reduce local recurrence rates of tumors and improve the survival and prognostic quality of life.

However, it has been shown that breast cancer patients often experience significant physical and mental stress responses(e.g., anxiety and depression) and functional adaptation disorders postoperatively along with low self-efficacy and negative coping styles(e.g., avoidance and compromise), which can affect their recovery process and prognostic quality of life.^5^ Conventional postoperative care often focuses on the physiological recovery of breast cancer patients, with insufficient intervention in their mental adaptation. Therefore, breast cancer patients frequently face dual challenges to their postoperative physical and mental health.^6^ To promote the comprehensive postoperative recovery of breast cancer patients, clinical attention and intervention regarding their mental adaptation need to be strengthened, thereby enhancing their quality of life and achieving overall physical and mental rehabilitation. In this regard, mindfulness-based complementary alternative therapy boasts non-pharmaceutical, low-risk characteristics and can regulate patients’ postoperative physical and mental stress responses while promoting psychological adaptation through the integration of mindfulness meditation, mind-body training, and cognitive restructuring.^7^

Currently, however, the research on the application of mindfulness-based complementary alternative therapy in the population of postoperative breast cancer patients remains limited. Therefore, this study was designed to explore the intervention efficacy of mindfulness-based complementary alternative therapy on the physical and mental stress, self-efficacy, and coping styles of patients undergoing breast cancer surgery, so as to provide evidence-based support for psychological care in clinical practice.

## METHODOLOGY

This was a retrospective study. A total of 80 patients who underwent surgical treatment in the Department of Breast Surgery at Hefei Cancer Hospital Breast Cancer Center, Chinese Academy of Sciences from May 2023 to December 2024 were enrolled for this study. Patient data including demographic data, diagnosis of breast carcinoma, were retrieved from electronic medical record systems, who were pathologically confirmed as breast cancer postoperatively and were divided into an intervention group(n = 40) and a control group(n = 40) using a randomized controlled approach, with no significant differences in clinical data between the two groups(P> 0.05).

### Ethical approval:

The study was approved by the Institutional Ethics Committee of Hefei Cancer Hospital, Chinese Academy of Sciences (No.:PJ-KY2024-035; date: July 12, 2024), and written informed consent was obtained from all participants.

### Inclusion criteria:

- Patients who underwent radical mastectomy or breast-conserving surgery at our hospital and pathologically confirmed as breast cancer.
- Participants who only engaged in this study during the same period.
- Patients with clear consciousness and without severe cognitive impairment.
- Age ≥ 18 years.
- II-III stage.

### Exclusion criteria:

- Patients who withdrew from the study or were lost to follow-up.
- Those who had previously undergone systematic mindfulness training.
- Those with communication barriers.
- Those with severe organ failure, such as renal failure.
- Patients with acute illness, lactic acidosis and connective tissue disorders.

### Interventions

The nursing measures for the control group strictly followed the “Guidelines for the Diagnosis and Treatment of Breast Cancer”^8^ and standard postoperative care protocols, covering standard care such as physiological function recovery, complication prevention, and basic mental support. Meanwhile, the intervention group was provided with mindfulness-based complementary alternative therapy integrated into the measures for the control group as postoperative care. Specific measures were as follows:

- Group sessions were organized twice a week (8-10 people per group) to lead patients in mindfulness meditation, guiding them to sit quietly with closed eyes and perceive bodily sensations from the feet to the head. Emphasis was placed on observing tension or numbness in the incision area, along with body acceptance training. Patients were guided to describe their pain with a “non-judgmental” attitude (e.g., describing it as a burning sensation rather than unbearable pain). The above process was implemented as daily home practice (15-min audio guidance sent via WeChat);
- Postoperative patients, as permitted by their physical condition, participated in unified classes for mindfulness yoga, with some poses supported by yoga straps. Poses included Cat-Cow, Tree Pose, and Supine Butterfly, among others;
- Patients were assisted in cognitive behavioral restructuring with themes including redefining the meaning of illness and positively transforming coping strategies. For example, “Breast cancer is a life challenge, not a punishment” and “I cannot endure chemotherapy” was reframed to “I can gradually adapt to treatment.” Patients were also encouraged to record one positive experience daily;
- Peer support groups were established (monthly offline activities) to share mindfulness practice experiences. The total duration of interventions lasted eight weeks.

### Observation indicators:

*General Baseline Data:* General baseline data for both groups, including age, body mass index (BMI), education level, marital status, disease stage, and surgical methods, were collected for comparison. Physical and Mental Stress: Anxiety and depression symptoms were assessed in both groups at two time points pre- and post-intervention (week eight) using the Self-Rating Anxiety Scale (SAS) and the Self-Rating Depression Scale (SDS),^9^ with a score of 50/53 indicating anxiety and depression, where higher scores suggest worse psychological states. Meanwhile, the postoperative pain levels were assessed with the Visual Analog Scale (VAS),^10^ providing a quantitative analysis of pain management. The VAS scale ranges from 0 to 10 cm, representing no pain to severe pain, with higher scores indicating more severe pain. Postoperative Self-Efficacy of Patients: The patient’s self-efficacy levels pre-intervention and at week eight post-intervention were assessed using the General Self-Efficacy Scale (GSES),^11^ which contains 10 items and adopts a 4-point Likert scale (1-4 points), with a total score of 10-40 points, where higher scores correlate positively with self-efficacy.

The reliability and validity of the scale have been validated, showing that its internal consistency reliability (Cronbach’s α = 0.87) and test-retest reliability (r = 0.83) met psychometric criteria. Postoperative Coping Styles of Patients: The coping styles of both groups pre-intervention and at week 8 post-intervention were assessed using the Medical Coping Modes Questionnaire (MCMQ),^12^ which contains three dimensions: Confrontation (eight items), Avoidance (seven items), and Compromise (five items), with a total of 20 items. It employs a 4-point Likert scale (1-4 points), with the score for each dimension being the sum of the corresponding item scores, where a higher score indicates a higher frequency of use of that coping style. This scale has demonstrated good reliability and validity in cancer patient populations (Cronbach’s α coefficients: Confrontation = 0.78, Avoidance = 0.72, Compromise = 0.68). Patient Satisfaction: Eight weeks post-intervention, the intervention satisfaction of both groups was evaluated using a self-made satisfaction scale from our hospital, with a total score of 95 points and ≥76 points for Satisfied, 57-75 points for Somewhat Satisfied, and <57 points for Dissatisfied. The intervention satisfaction score is calculated as Satisfied + Somewhat Satisfied.

### Quality control:

In this study, a questionnaire survey method was utilized, with investigators from our hospital distributing the scales and uniformly trained investigators guiding respondents to complete the questionnaires on-site while providing explanations as needed. After collection, the completeness of the questionnaires was checked, with any missing items promptly supplemented. During data processing, the data were entered by two individuals on two separate computers, with a logical error rate of <0.1%. Additionally, data analysts did not participate in the implementation of interventions, so as to reduce bias.

### Statistical methods:

SPSS 25.0 software was used to test for statistical differences. Measurement data were expressed as *“X̄±S“*, with t-tests conducted. Count data were expressed as cases (%), and χ^2^ tests were performed. *P< 0.05* was considered statistically significant.

## RESULTS

The comparison of clinical data between the two groups showed no statistically significant differences (*P > 0.05*), as shown in Table-I. After intervention, SAS, SDS, and VAS scores in the intervention group were lower than in the control group (*P < 0.05*), as shown in Table-II. Post-intervention GSES scores in the intervention group were higher than in the control group *(P < 0.05*), as shown in Table-III.

**Table-I:** Comparison of general baseline data between the 2 groups [*(X̄±S)*, n (%)].

Indicator	Intervention Group (n = 40)	Control Group (n = 40)	t/value	P value
Age (years)	46.63 ± 9.78	47.25 ± 8.24	0.307	0.760
BMI (kg/m²)	22.24 ± 1.61	22.36 ± 1.54	0.341	0.734
Education Level			0.202	0.653
Middle school or below	19 (47.50)	17 (42.50)		
Above middle school	21 (52.50)	23 (57.50)		
Marital Status			0.738	0.390
Married	34 (85.00)	31 (77.50)		
Unmarried	6 (15.00)	9 (22.50)		
Disease Stage			0.050	0.823
Ⅱ stage	18 (45.00)	19 (47.50)		
Ⅲ stage	22 (55.00)	21 (52.50)		
Surgical Method			0.056	0.813
Breast-conserving surgery	14 (35.00)	13 (32.50)		
Radical mastectomy	26 (65.00)	27 (67.50)		

**Table-II:** Comparison of Physical and Mental Stress between the 2 Groups [points, *(X̄±S)*].

Group	n	SAS		SDS		VAS	
		Pre-intervention	Post-intervention	Pre-intervention	Post-intervention	Pre-intervention	Post-intervention
Intervention	40	43.38 ± 5.78	20.87 ± 4.69	44.63 ± 6.75	22.12 ± 3.16	6.81 ± 1.05	2.58 ± 0.54
Control	40	43.13 ± 5.56	30.73 ± 4.85	44.26 ± 6.58	31.45 ± 3.78	6.76 ± 1.14	4.06 ± 0.37
*t* value		0.197	9.243	0.248	11.977	0.204	14.299
*P* value		0.844	<0.001	0.805	<0.001	0.839	<0.001

***Note:*** Compared with the pre-intervention values in the same group, *P < 0.05.

**Table-III:** Comparison of pre- and post- intervention GSES scores between the 2 Groups [points, *(X̄±S)*].

Group	n	GSES scores	
		Pre-intervention	Post-intervention
Intervention	40	18.81 ± 3.54	31.15 ± 5.16*
Control	40	18.63 ± 2.94	25.31 ± 4.28*
*t value*		0.247	5.509
*P value*		0.805	<0.001

***Note:*** Compared with the pre-intervention values in the same group,

* P < 0.05.

After intervention, the intervention group exhibited a higher “Confrontation” score and lower “Avoidance” and “Compromise” scores than the control group (*P < 0.05*), as shown in Table-IV. The overall satisfaction of 97.50% in the intervention group was higher than the 85.00% in the control group (*P < 0.05*), as shown in Table-V.

**Table-IV:** The comparison of postoperative coping styles between the 2 groups [points, *(X̄±S)*].

Group	n	Confrontation		Avoidance		Compromise	
		Pre-intervention	Post-intervention	Pre-intervention	Post-intervention	Pre-intervention	Post-intervention
Intervention	40	13.86 ± 2.84	20.87 ± 4.45*	16.54 ± 3.87	10.68 ± 2.07*	8.58 ± 2.49	4.78 ± 1.57*
Control	40	13.54 ± 2.75	17.39 ± 3.28*	16.41 ± 3.17	13.54 ± 2.21*	8.64 ± 2.16	6.37 ± 1.42*
*t* value		0.512	3.981	0.164	5.974	0.115	4.750
*P* value		0.610	<0.001	0.870	<0.001	0.909	<0.001

***Note:*** Compared with the pre-intervention values in the same group,

* P < 0.05.

**Table-V:** Comparison of overall satisfaction between the 2 groups [n (%)].

Group	n	Satisfied	Somewhat Satisfied	Dissatisfied	Overall Satisfaction
Intervention	40	22 (55.00)	17 (42.50)	1 (2.50)	39 (97.50)
Control	40	20 (50.00)	14 (35.00)	6 (15.00)	34 (85.00)
*χ^2^value*					3.914
*P value*					0.048

## DISCUSSION

The findings of this study showed a decrease in SAS, SDS, and VAS scores in both groups eight weeks post-intervention, with lower SAS, SDS, and VAS scores in the intervention group than in the control group, indicating that an eight weeks mindfulness-based complementary alternative therapy can effectively alleviate postoperative physical and mental stress responses in patients undergoing breast cancer surgery. The possible reasons behind this may include that mindfulness-based complementary alternative therapy helps patients disengage from fears about the future or entanglements with the past through the training of “present-moment awareness”, thereby reducing rumination. Particularly, mindfulness guides patients to observe rather than confront negative emotions (e.g., anger and sadness), enhancing their emotional regulation abilities.^13^-^15^ Additionally, mindfulness meditation and body awareness can distract from pain and regulate pain-related brain regions, alleviating postoperative pain or neuropathic pain caused by chemotherapy.^16^ Therefore, eight weeks of mindfulness-based complementary alternative therapy for patients undergoing breast cancer surgery can effectively alleviate their postoperative physical and mental stress responses.

At the same time, the findings also suggested an increase in GSES scores in both groups eight weeks post-intervention, with the intervention group showing higher GSES scores than the control group. Additionally, the “Confrontation” score increased, while the “Avoidance” and “Compromise” scores decreased in both groups, with the intervention group exhibiting a higher “Confrontation” score and lower “Avoidance” and “Compromise” scores compared to the control group after eight weeks of intervention, indicating that 8 weeks of mindfulness-based complementary alternative therapy for patients undergoing breast cancer surgery can enhance their sense of self-efficacy and optimize their coping strategies. This is possibly due to the fact that mindfulness-based complementary alternative therapy empowers patients with tools for actively managing their emotions, assists in cognitive-behavioral restructuring, and enhances their sense of control over life, thereby improving self-efficacy and alleviating feelings of helplessness.^17^

Moreover, group mindfulness courses provide a safe space for emotional expression, reduce feelings of loneliness, and enhance social belonging, with long-term mindfulness practice also promoting functional connectivity in brain regions associated with emotional regulation, ultimately enhancing psychological adaptability and promoting neural plasticity.^18^ Therefore, eight weeks of mindfulness-based complementary alternative therapy for patients undergoing breast cancer surgery can enhance their sense of self-efficacy and optimize their coping strategies.

Furthermore, the findings also revealed an overall satisfaction rate of 97.50% in the intervention group, higher than 85.00% in the control group, indicating high patient satisfaction with the eight weeks mindfulness-based complementary alternative therapy for patients undergoing breast cancer surgery. The reasons for this may be that the mindfulness-based complementary alternative therapy helps patients observe bodily changes with a non-judgmental attitude, cope with changes in body image, and mitigate feelings of shame or self-denial related to breast loss, thereby promoting psychological adaptation.^19^ Additionally, mindfulness breathing and yoga can help manage side effects from treatment (e.g., nausea, hot flashes, or insomnia), partially alleviating physical discomfort.^20^ Given this, eight weeks of mindfulness-based complementary alternative therapy for patients undergoing breast cancer surgery results in high satisfaction.

### Limitations

It has a small sample size and being a single-center study. In view of this, large-sample, multi-center studies should be performed in future research, aiming to further validate the usefulness of mindfulness-based complementary alternative therapy in postoperative care for breast cancer patients.

## CONCLUSIONS

Eight weeks of mindfulness-based complementary alternative therapy for patients undergoing breast cancer surgery can effectively alleviate postoperative stress responses, enhance self-efficacy, and optimize coping strategies, demonstrating promotional value in clinical practice.

### Authors’ Contributions:

**YW:** Conceptualization, design, literature search, data collection, analysis, interpretation and critical review.

**ZD:** Investigation, writing, critical review and editing.

All authors have read and approved the final manuscript and are responsible for integrity of the study.
